# Modulators of radiation-induced cardiopulmonary toxicities for non-small cell lung cancer: Integrated cytokines, single nucleotide variants, and HBP systems imaging

**DOI:** 10.3389/fonc.2022.984364

**Published:** 2022-12-15

**Authors:** Yuki Mukai-Sasaki, Zhongxing Liao, David Yang, Tomio Inoue

**Affiliations:** ^1^ Department of Radiation Oncology, The University of Texas MD Anderson Cancer Center, Houston, TX, United States; ^2^ Advanced Medical Center, Shonan Kamakura General Hospital, Kamakura, Japan

**Keywords:** radiation therapy, carditis, pneumonitis, glucosamine imaging, cytokine, SNV, SNP

## Abstract

Radiation therapy (RT)-induced cardiopulmonary toxicities remain dose-limiting toxicities for patients receiving radiation dosages to the thorax, especially for lung cancer. Means of monitoring and predicting for those receiving RT or concurrent chemoradiation therapy before treatment begins in individual patients could benefit early intervention to prevent or minimize RT-induced side effects. Another aspect of an individual’s susceptibility to the adverse effects of thoracic irradiation is the immune system as reflected by phenotypic factors (patterns of cytokine expressions), genotypic factors (single nucleotide variants SNVs; formerly single nucleotide polymorphisms [SNPs]), and aspects of quantitative cellular imaging. Levels of transcription, production, and functional activity of cytokines are often influenced by SNVs that affect coding regions in the promoter or regulatory regions of cytokine genes. SNVs can also lead to changes in the expression of the inflammatory cytokines, interferons, interleukins (IL-6, IL-17) and tumor necrosis factors (TNF-α) at the protein level. RT-induced cardiopulmonary toxicities could be quantified by the uptake of ^18^F-fluorodeoxyglucose (FDG), however, FDG is a sensitive but not specific biomarker in differential diagnosis between inflammation/infection and tumor recurrence. FDG is suitable for initial diagnosis of predisposed tissue injuries in non-small cell lung cancer (NSCLC). ^99m^Tc-ethylenedicysteine-glucosamine (^99m^Tc-EC-G) was able to measure tumor DNA proliferation and myocardial ischemia *via* hexosamine biosynthetic pathways (HBP). Thus, ^99m^Tc-EC-G could be an alternative to FDG in the assessment of RT doses and select patients in HBP-directed targets for optimal outcomes. This article reviewed correlative analyses of pro-inflammatory cytokines, genotype SNVs, and cellular imaging to improve the diagnosis, prognosis, monitoring, and prediction of RT-induced cardiopulmonary toxicities in NSCLC.

## 1 Radiation therapy modalities for lung cancer

Lung cancer is the most common cancer worldwide and the second most commonly diagnosed cancer in both men and women in the United States. The most prevalent subtype is NSCLC; a small-cell lung cancer variant (SCLC) is less common but deadly because of its propensity to metastasize, especially to the brain, before it is detected. Radiation therapy (RT) is a key component of both definitive and palliative therapy for lung cancer. In NSCLC, RT can be used to shrink tumors before surgery, or to eliminate residual tumor cells after surgery. In SCLC, prophylactic cranial irradiation helps to suppress or delay metastatic tumor to the brain.

In the past, conventional radiation treatments were based on 2-dimensional (2D) X-ray images, a practice that resulted in suboptimal dose distributions to both the target and surrounding normal structures. Advances in external-beam radiation therapy since the era of 2D-based therapy include stereotactic ablative (body) RT (SABR or SBRT), 3D conformal RT, intensity-modulated (IM) RT, volumetric modulated arc therapy (VMAT), and stereotactic radiosurgery (SRS). Each of these advanced techniques requires the use of sophisticated 3D- and sometimes 4D-imaging systems. SBRT uses tumor tracking and image guidance to enable the delivery of high-dose radiation to the tumor while sparing normal tissues, and has shown particular promise for early-stage lung cancer. In 3D conformal RT, tumor locations are mapped precisely through the use of computed tomography (CT) scans, and radiation beams are shaped and aimed at the tumor from several directions, which reduces the likelihood of damaging nearby normal tissues. In IMRT, radiation beams are also shaped and aimed at the tumor from several angles, but the intensity (strength) of the beams can also be adjusted to limit the dose that reaches normal tissues. VMAT is similar to IMRT but radiation is delivered in arcs through rotation of the linear accelerator, which can speed the treatment process. IMRT or VMAT are often used for tumors that are near important structures such as the spinal cord. SRS is used to treat intracranial tumors. SRS also uses multiple beams of radiation aimed at tumors in the brain from different angles, and is delivered over a few minutes to hours. Use of the Cyberknife imaging guidance system or novel tomotherapy systems that combine dynamic helical radiotherapy with imaging guidance can enable highly effective and accurate stereotactic radiation delivery (1). Collectively, these advanced techniques can optimize outcomes in tumor control, minimize normal tissue damage, and prolong survival for patients with lung cancer.

Another advancement in RT is the use of proton beam therapy, which also enables precise targeting of tumors with greatly reduced radiation exposure beyond the tumor target. The enhanced ability to avoid normal tissue exposure from proton therapy is thought to minimize toxic reactions and thereby improve patients’ quality of life relative to X-ray (photon) therapy. However, proton therapy is subject to physical uncertainties associated with the ability to target tumors consistently between fractions, particularly tumors that move with respiration or cardiac function, and to account for inconsistencies in patient set-up and positioning between treatments. At present, proton therapy is more expensive than conventional IMRT or VMAT methods. RT, whether delivered by IMRT or VMAT, or as proton therapy, is also effective in relieving symptoms caused by more advanced lung tumors such as pain, bleeding, or airway blockage. Regardless of whether the intent of treatment is curative or palliative, the goals of external-beam radiation therapy are the precise delivery of high-dose radiation to targeted areas while minimizing the dose to surrounding tissues.

## 2 Management of radiation-induced cardiopulmonary toxicities

### 2.1 Pneumonitis

Although technologic advances have considerably improved the ability to deliver targeted radiation for the treatment of NSCLC and other thoracic tumors, RT nevertheless may induce pneumonitis when the radiation doses are high or when large volumes of lung tissue are exposed (2–7). One approach to establish safe radiation dose limits for healthy lung tissue was to include mathematical models of normal tissue complication probability. In this approach, dose-volume histogram metrics are used to derive radiation dose limits. One such limitation in common use is the mean lung dose (8). However, the relationship between mean lung dose and risk of radiation pneumonitis is also influenced by factors such as an individual’s response to chemotherapy, heterogeneity in the composition of the tumor, and tumor location (9–11). Therefore, these models are not ideal for predicting which individual patients will develop pneumonitis in response to radiation.

To monitor and predict the development of pneumonitis by RT in individuals, attempts have been incorporated with other physiological factors such as circulating levels of immune-related cytokine patterns (e.g., interleukins [ILs], interferons [IFNs], tumor necrosis factor [TNF], and transforming growth factor [TGF]). All of these biomarkers have been linked with the risk of radiation pneumonitis (12, 13). Other genotypic factors such as SNVs (previously known as SNPs), particularly those in pathways involved in DNA repair, cell cycling, tumor necrosis, and angiogenesis have also been tested for their ability to predict radiation pneumonitis in individual patients (14, 15). Another reported model incorporates both dose-volume component and genetic component (SNVs) in further attempt to optimize lung dosage for individual patients being treated for NSCLC (11). The reports in genotype factors suggested that predictions of pneumonitis could be improved by using this combination of components relative to mathematic models on dose-volume effects.

RT-induced pneumonitis occurs in three phases: (i) ultrastructural changes in the alveolar–capillary barrier and alveolar epithelial cells at the molecular and cellular levels (microvascular permeability, interstitial edema, apoptosis), occurring during the first 2-4 weeks after irradiation; (ii) inflammation triggered by cascades of cytokines and expression of hypoxic genes that damage the lung parenchyma, epithelial cells, vascular endothelial cells, and stroma, occurring from 1-6 months after irradiation (i.e., the acute phase); and (iii) lung fibrosis, occurring more than 6 months after irradiation (i.e., the chronic phase) (16, 17).The most common symptoms of pneumonitis are shortness of breath, fatigue, weight loss, loss of appetite, and dry cough, which may become chronic. Chronic pneumonitis can lead to permanent pulmonary fibrosis, heart failure, and death. Corticosteroids and oxygen therapy are used to reduce inflammatory cell infiltration and cytokine expression of TNF-α, IL-6, IL-17A, and TGF-β1 in broncho-alveolar lavage fluid in radiation-induced pneumonitis (18). In patients who cannot tolerate steroids or are unresponsive, investigators reported the use of radiation protectors, modifiers, or mitigators for acute or late responding normal tissues, ideally without any protective effect on the tumor cells. An example of amifostine, a radioprotector, was given before radiation exposure making normal cells more resistant to permanent RT-induced DNA damage by neutralizing reactive oxygen species (ROS) and increasing oxygen consumption in normal cells. Radiomitigators were given during or immediately after radiotherapy but before the appearance of toxicity. For instance, methyl prednisone, an immune suppressor, could reduce TGF-β1 and TNF-α. Lovastatin and ulinastatin (HMG-co-reductase inhibitors) could enhance the concentration of endothelial nitric oxide synthase which promotes anti-inflammatory signaling, prevents apoptosis, increases vasodilatation, and reduces platelet adhesion. The Angiotensin II and renin-angiotensin-system agents (Captopril, Enalapril) were used for mitigating late radiation effects. These drugs modulate the effects of TGF-β in radiation damage by targeting the oxidant, inflammatory and fibrogenic pathways (19). Others reported the use of an anti-oxidant (curcumin, genistein) and Keratinocyte Growth Factor (KGF) to stimulate the proliferation and differentiation of alveolar type 2 cell and protects the RT-induced lung inflammation and fibrosis. After the appearance of toxicity, bone marrow derived mesenchymal stem cell therapy could be administered to modulate progression or reverse the damage (20, 21).

Exposure of the heart to radiation during treatment has also been examined for possible relationship with the development of pneumonitis (22–26). Some, but not all, of these reports suggested that minimizing the dose to the heart (“dose sparing”), particularly the upper regions of the heart, could improve RT-induced pneumonitis. One example involved evaluating the relationship between radiation doses to the heart and its substructures and pneumonitis during treatment for thymic epithelial tumors, because these tumors generally originate within the anterior mediastinal region and can spread into the pericardial space (25). This retrospective analysis indicated that RT-induced pneumonitis appeared despite the mean lung dose and lung V20 being lower than those used for locally advanced NSCLC, and that this seemed to be related to the dose (V35) to the pulmonary artery; the authors proposed that minimizing the dose to the pulmonary artery might reduce the incidence of pneumonitis in patients undergoing mediastinal RT. Concurrent chemoradiation therapy, particularly with bleomycin, cyclophosphamide, carmustine, and etoposide, has also been reported to increase the risk of pneumonitis for SCLC (27, 28).

### 2.2 Carditis

As noted in the previous section, because locally advanced lung cancer can involve the mediastinal lymph nodes that lie behind the heart, some exposure of the heart may be unavoidable during RT for lung cancer. Exposure of the heart’s three layers (pericardium, myocardium, and endocardium) can lead to three corresponding types of carditis (pericarditis, myocarditis, and endocarditis). The pericardium is the outermost layer of the heart and contains nerves and blood vessels that support the heart. The myocardium is the muscle of the heart, which enables the heart to contract during systole and diastole. The endocardium is the inner layer of the heart, and consists of connective tissue linked to the inner surfaces of the heart chambers and valves. In decreasing order of occurrence, RT-induced cardiac toxicities can include ischemic and infarction myocarditis, pericardial toxicity, valvular toxicity, or arrhythmias. “Myocarditis” is a general term to describe inflammation of the myocardium; the ‘gold standard’ for diagnosing myocarditis (and determining its etiology) is endomyocardial biopsy, which is used infrequently because of its invasive nature. According to the World Health Organization, other means of diagnosing myocarditis include histological, immunological, immunohistochemical, and molecular analyses; the diagnosis may also be made based on circumstantial evidence such as abnormalities on echocardiography or magnetic resonance imaging (MRI). In patients with NSCLC, myocarditis is generally diagnosed on the basis of biomarkers (troponin, CPK, myoglobulin), immunohistopathologic findings, and imaging findings. For instance, cardiotoxicity can be defined by analyzing the change in the left ventricular shortening fraction (LVSF) diagnosed *via* ECHO, or as a clinical diagnosis showing apparent heart failure in the patient. If LVSF is lower than 28%, or if there is more than 10% change in LVSF from baseline, this constitutes cardiotoxicity (29).

### 2.3 ROS-mediated DNA damage in pulmonary fibrosis and carditis

**Figure 1:**
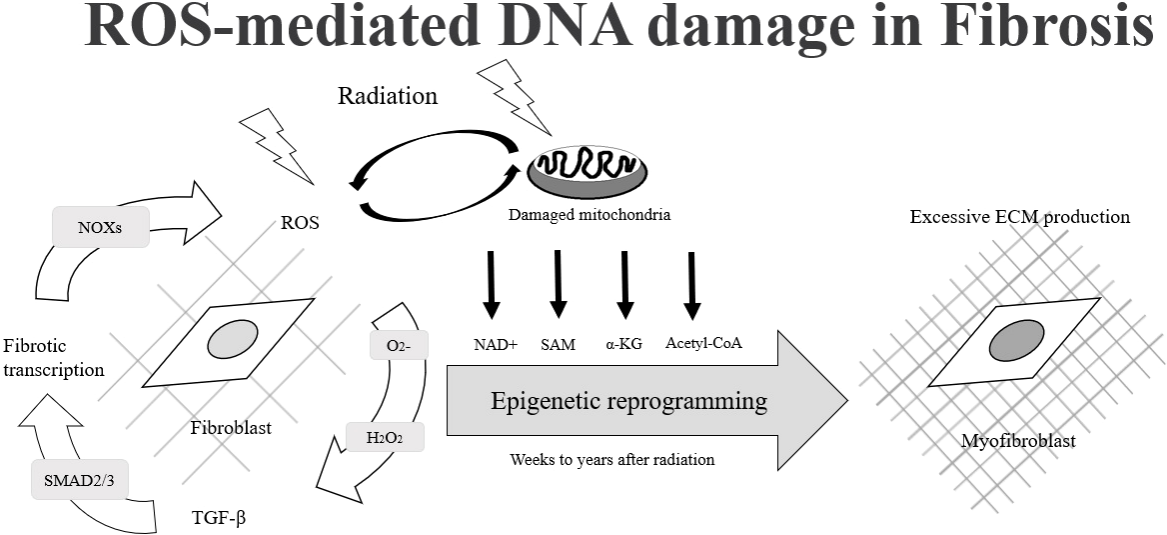
The process of fibroblast cells changes to myofibroblast cells due to ROS-mediated DNA damage.

**Figure 2:**
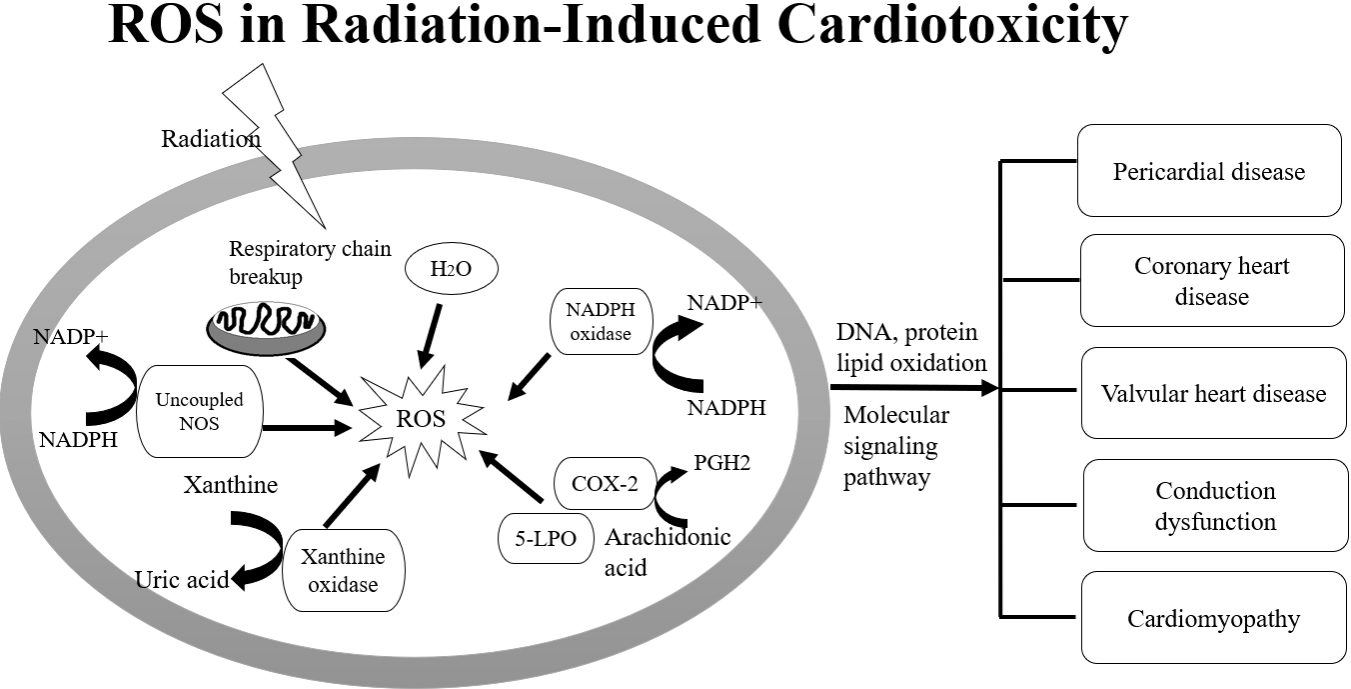
Imbalance between ROS, NOS and up-regulation of NADPH oxidases are involved in DNA damage in carditis after irradiation.

Most of the radiation damage (X-ray, gamma rays, rapid electrons) after exposure to radiation is caused by the generation of reactive oxygen species (ROS) and reactive nitrogen species (RNS). ROS and RNS are produced by the radiation decomposition of water, which is an important source of normal tissue damage after ionizing radiation. ROS plays an important role in radiation-induced epigenetic changes and causes differentiation of normal fibroblasts to myofibroblasts. Under normal conditions, myofibroblasts are responsible for normal wound closure after injury. After wound healing and restoration of extracellular matrix (ECM) to homeostatic levels, the myofibroblasts undergo apoptosis. If the myofibroblasts do not undergo apoptosis after healing and continue to damage, then aberrant amounts of extracellular matrix proteins may occur and cause fibrosis (**Figures 1**, **2**) (30, 31). The degranulation of mast cells triggered by ROS generation, complement activation, cytokine stimulation or adenosine after irradiation may lead to the release of several mast cell-derived fibrogenic mediators, such as TNF-α, TGF-β, IL-4 and PDGFs, which may trigger or amplify the fibrotic response. Up-regulated TGF-β1 at the protein and the mRNA level is one of the most effective initiators for inducing differentiation of myofibroblasts from resident fibroblasts and bone marrow progenitors (32). Activated myofibroblasts can enhance the synthesis of ECM protein, increase the expression of integrins and suppress matrix metalloproteinases. Abnormal accumulation of ECM separating and/or replacing myocytes increases ventricular stiffness, decreases elasticity and distensibility and can lead to contractile dysfunction. Excess fibroblasts and ECM can cause damage to the mechano-electric coupling of cardiomyocytes, thus decreasing cardiac contraction and raising the risk of arrhythmogenesis and mortality. Besides, inflammation and fibrosis within perivascular regions may cause a reduction in tissue utilization of oxygen and nutrients and increase the adverse cardiac remodeling. These factors ultimately lead to decreased elasticity and distensibility, thereby resulting in reduced ejection fraction and cardiac failure (32–34). ROS also acts as a second messenger to alter the expression of multiple proteomes in the cytoplasm. Sallam M, et al., provided a summary of the ionizing radiation in epigenetic modifications, especially DNA methylation alterations in carditis. The activation of NF-κB induces the production of inflammatory cytokines. NF-κB regulates DNA transcription and protein complexes engage in various cellular oxidative stress responses. The change the integrity of the genome could lead to cell cycle arrest, apoptosis, mutation and other effects (35).

Wang B et al., summarized the underlying pathophysiology involved in the initiation and progression of radiation-induced myocardial fibrosis. They reported the imbalance between ROS and nitric oxide (NO) as well as up-regulation of NADPH oxidases are responsible for vascular injury after irradiation. Vascular injury and endothelial dysfunction can promote the overproduction of oxygen free radicals and induce redox reactions (36). During vascular injury, chronic hypoxia and the expression of hypoxia-inducible factor-1 (HIF-1α) can trigger angiogenesis by up-regulating the expression of vascular endothelial growth factor (VEGF) and TGF-β, as well as the induction of inflammatory mediators. Injured endothelial cells can also increase the capacity for monocyte adhesion through inflammatory adhesion molecules such as vascular cell adhesion molecule 1 (VCAM-1), intercellular adhesion molecule-1 (ICAM-1) and E-selectin. Invading monocytes are transformed into activated macrophages and recruited into the intima *via* monocyte chemotactic protein-1 to form fatty streaks in the presence of elevated cholesterol. Monocytes absorb low-density lipoprotein to form lipid-laden foam cell aggregates after transmigration, which further secrete inflammatory cytokines and pro-fibrotic chemokines such as TGF-β, stimulate the differentiation of smooth muscle cells into myofibroblasts, and generate large quantities of type IV collagen, eventually leading to arterial stenosis and fibrosis (36). To prevent RT-induced microvascular damage, inflammation, and late fibrosis in pulmonary and heart, some drugs including statins, angiotensin-converting enzyme inhibitors, and antioxidants were used as therapeutic strategy to treat cellular targets (33, 36).

## 3 Modulators of radiation-induced pulmonary toxicities

### 3.1 Immune cells and fibroblasts in radiation-induced lung fibrosis and carditis

**Figure 3:**
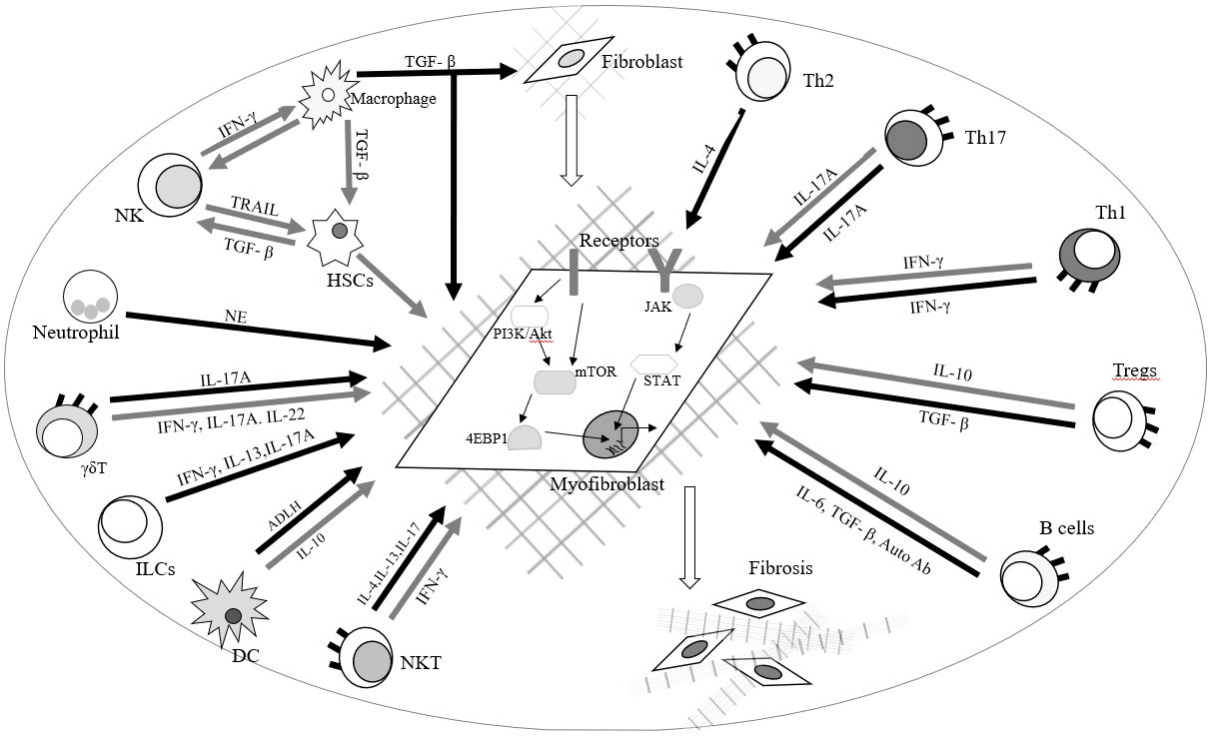
Roles of immune cells and fibroblasts in radiation-induced lung fibrosis and carditis.

Radiation-induced lung fibrosis and carditis are potentially lethal clinical complication during chest RT. Pulmonary fibrosis can lead to distorted pulmonary architecture, impaired lung function, and alveolar gas exchange, resulting in hypoxemia, dyspnea and exercise intolerance. Myocardial fibrosis in carditis is characterized by decreased ventricular elasticity and distensibility, which can result in decreased ejection fraction, heart failure and even sudden cardiac death. Huang E et al., summarized recent advances in understanding the roles of immune cells in regulating fibrotic development and immune-based therapies in different disorders. During inflammatory responses, activated immune cells orchestrate the cellular and molecular processes of fibrosis in responses to external stimuli and microenvironmental factors. The recruitment and activation of immune cells including macrophages, neutrophils, natural killer (NK) cells, T cells, and B cells regulate the progression and regression of fibroblast production in various organs and tissues through different molecular mechanisms. The authors also reported the molecular mechanisms with a focus on mTOR and JAK-STAT signaling pathways (**Figure 3**) (37). Wang B et al., reported that the radiation-induced vascular endothelial injuries induce the inflammatory chemokine secretion and degradation of the endothelial basement membrane by matrix metalloproteinases to recruit leucocytes to injured sites. Neutrophils migrate to damaged sites, mediated by adhesion molecules such as E-selectin, ICAM-1 and VCAM-1, and secrete growth factors and inflammatory mediators including TNF, IL-1, IL-6 and IL-8, to mediate the acute inflammation response. These mediators also release pro-fibrotic cytokines like platelet-derived growth factor (PDGF), insulin-like growth factor (IGF), basic fibroblast growth factor (FGF), TGF-β and CTGF to promote ROS development leading to chronic inflammation (36). The multiple TGF-β, ILs, INFs, TNFs, and cytokines amplify the inflammatory response and trigger the recruitment and proliferation of fibroblasts (36, 38–40). Subsequently, interactions between monocytes and lymphocytes promote the differentiation of monocytes into M1 and M2 macrophage subsets. Increased M2 macrophages enhance proliferation and differentiation of fibroblasts into myofibroblasts by the secretion of TGF-β and FGF at the injured site. Increased myofibroblasts produce large quantities of type IV collagen, eventually leading to fibrosis and cell death (36).

### 3.2 IL-6 and IL-17

RT-induced carditis, pneumonitis and fibrosis remain the main limiting factors for efficient RT. Variations in the susceptibility to RT-induced cardiopulmonary toxicities after ionizing radiation among individuals depend on genetic makeup as well as radiation doses (41). For example, single-fraction RT doses of <7.5 Gy led to near-0% incidence of pneumonitis in humans, but that incidence rose to 50% after a single-fraction dose of 9.3 Gy. During ionizing radiation, activation of various signal transduction pathways induces processes that lead to replacement of damaged cells, influx of inflammatory cells from peripheral blood, and production of cytokines that influence proliferation, chemotaxis, mediation of inflammatory responses, homeostasis, differentiation, and cell death (38).

Cytokines have active roles during all three phases of RT-induced pneumonitis as well as in carditis. Cytokines are involved in cell signaling in pathological processes, but levels of cytokines can also be useful in diagnosis. Some have posited that a mechanism underlying radiation-induced cardiopulmonary toxicity is hyperactivation of the immune system, characterized by the release of numerous inflammatory mediators, including ILs, INFs, TNFs, and cytokines (16, 39, 40, 42). Circulating levels of these factors that exceed normal thresholds can prompt cytokine release syndrome, a systemic inflammatory process that leads to massive cytokine production by proliferating activated T cells. IL-6 is the principal mediator in the development of cytokine release syndrome in addition to IL-2, IL-8, IL-10, IL-17, INFγ, and TNFα. The circulating levels of these cytokines are thought to be correlated to the severity of the syndrome. Cytokine release syndrome has been linked with life-threatening cardiovascular complications including hypotension, shock, tachycardia, arrhythmias (ranging from asymptomatic prolonged corrected QT interval to supraventricular tachycardia, atrial fibrillation, flutter, and ventricular arrhythmias (e.g., Torsade de Pointes), left ventricular dysfunction, heart failure, and cardiovascular death. Another proposed mechanism for radiation-induced cardiopulmonary toxicity is the formation of microvascular and macrovascular thrombi that result from the radiation-induced activation of platelets, neutrophils, and other proteins, which can contribute to vascular occlusion by microvascular thrombosis and myocardial infarction (43, 44). Further understanding of how cytokines that affect inflammatory processes affect RT-induced cardiopulmonary toxicity (and vice versa) may improve the effectiveness of RT for cancer. The information available on potential correlations between the cytokines IL-6 and IL-17 and their responses to RT-induced cardiopulmonary toxicity was reviewed below.

Analysis of a panel of circulating cytokines with different putative functions in radiation pulmonary injury identified IL-17 and IL-6 as early circulating cytokine markers for radiation induced cardiopulmonary toxicities. TNF-α, is known to have its role in fibrosis development and leads to TGF-β1 induction. IL-6 is produced by mononuclear phagocytes, lymphocytes, keratinocytes, hepatocytes, bone marrow cells, alveolar macrophages, lung fibroblasts, and pneumocytes and in response to the production of various other cytokines (IL-1, IL-17 and TNF-α) (45). The key function of IL-6 is to stimulate the growth and differentiation of B and T lymphocytes, which activate inflammation and immune responses by driving the trafficking and activation of those lymphocytes and by inducing production of acute-phase proteins by hepatocytes. IL-6 promotes T-cell proliferation, B-cell differentiation and survival, and plasma cell production of IgG, IgA, and IgM. Binding of the IL-6 ligand to the IL-6 receptor transmembrane protein (gp130) triggers the Jak-STAT cascade [Janus kinase [Jak]-phosphorylated signal transduction and activator transcription protein [STAT]) and activates glucose and receptor tyrosine kinase signaling pathways such as PI3/AKT/mTOR and Ras/Raf/MAPK (46). Several groups have reported correlations between the occurrence of radiation-induced pneumonitis and cardiotoxicity and levels of IL-6 in patients during RT (39, 47, 48). In addition to their involvement in radiation-induced cardiopulmonary toxicity, TGF-β, IL-6, IL-1, and TNFα also regulate the release of IL-17 from Th17 cells. The development and proliferation of these T-helper cells and their secretion of IL-17 are regulated mainly by IL-6, TGF-β, and TNF-α (49). Although cytokine expressions of TNF-α, IL-6, IL-17A, and TGF-β1 are sensitive phenotyping biomarkers in radiation-induced cardiopulmonary toxicities, they are not specific in differentiation of inflammation/infection versus tumor recurrence. Thus, phenotyping biomarkers could produce false-positive findings. To overcome these issues, more specific quantitative genomic sequencings are amenable for modulating RT-induced cardiopulmonary toxicities.

### 3.3 SNVs

Levels of transcription, production, and functional activity of cytokines are often influenced by SNVs (formerly known as SNPs) that affect coding regions in the promoter or regulatory regions of cytokine genes. SNVs in certain alleles of pro-inflammatory cytokines can also increase susceptibility to inflammation. An example is the inflammatory IL-6; the presence of an SNV that codes for heterozygosity in the promotor region of the IL-6 gene has been linked with increased numbers of CpG sites, methylation events, and mRNA levels in inflammatory stress (15).

The use of SNV genotyping arrays was applied to identify common DNA variants present across the human genome. SNVs have been shown to be responsible for differences in genetic traits, susceptibility to disease, and response to drug therapies. Because of their abundance and presence throughout the entire human genome, SNVs have been widely used in genetic association studies of various complex diseases such as cancer, obesity, osteoporosis, asthma, and hypertension. The main advantage of SNV arrays in cancer is that DNA from tumor cells is used instead of that from mitotically dividing cells in cell cultures. Although the wide occurrence of SNVs in a gene or in a regulatory region generally act as biological markers for identified genes associated with important traits, SNV arrays also have limitations. SNV genotyping platforms often find that some SNV loci cannot be well detected and genotyped because the flanking regions of these SNVs are not conserved or there are other “hits” in the genome due to the sequence homology. The sensitivity causes many false-positive or false-negative results in SNV genotyping.

In addition to IL-6, IL-17, and SNVs, other biomarkers being considered as adjuncts in the diagnosis of pneumonitis include C-reactive protein, leukocyte count, immunoglobulins, and proinflammatory cytokines. Other biomarkers of growing interest include procalcitonin and TREM (triggering receptor expressed on myeloid cells). Deficiency or variations in IL-17, a cytokine mainly secreted by Th17 cells, can cause increased susceptibility to inflammation and infection by extracellular pathogens. For example, the rs2275913 SNV, located in the promoter area of the IL-17A gene, is associated with inflammation and infection; the presence of an A allele at the rs2275913 SNV increases the secretion of IL-17A. According to one meta-analysis, the IL-17A rs2275913 polymorphism is linked with the risk of many types of cancer (50). Another study showed that the rs2275913 SNV of *IL-17* is related to the severity of acute bronchiolitis and could lead to variations in IL-17 expression (51). Another group showed that patients with autoimmune diseases had increased frequencies of the heterozygous IL-6 promotor SNV rs1800795 (52). The presence of this functional SNV and its effects on cytokine patterns may be involved in radiation- or chemoradiation-induced cardiopulmonary inflammation. In addition to IL-6 and IL-17 levels, SNVs that affect cellular functions may be useful as biological markers, given that differences in their expression among patients has been linked with the incidence of radiation-induced toxicity (14, 15). SNVs have been shown to be responsible for differences in genetic traits, susceptibility to disease, and response to drugs and radiation. Analyses of SNV arrays can reveal genetic gains (duplications) or losses (deletions) that result in extra or missing copies of genetic material. Methods of detecting SNV sequences include allele-specific polymerase chain reaction (AS-PCR), restriction fragment length polymorphism (RFLP) PCR, TaqMan probes, multiplex PCR-based real-time invader assay (mPCR-RETINA), and next-generation sequencing. Apparently, quantitative analyses of SNVs are specific genotyping for RT-induced cardiopulmonary toxicities. The wide occurrence of SNVs in a gene or in a regulatory region generally act as biological markers for genes associated with important traits, SNV arrays also have limitations. Some SNV loci cannot be easily detected or genotypes on SNV genotyping platforms because the flanking regions of these SNPs are not conserved or other hits in the genome influence sequence homology, which can cause false-positive or false-negative results in SNV genotyping. In addition, both cytokine expressions and SNVs do not identify the location of cardiopulmonary toxicities as well as tumor status. Therefore, functional cellular imaging is an important factor to reduce the risk of developing severe radiation pneumonitis in interstitial lung disease and carditis based upon genotypes and phenotypes.

### 3.4 Cellular imaging as the modulator for radiation-induced cardiopulmonary toxicities

#### 3.4.1 Trends of hybrid (fused) imaging modalities in radiopharmaceutical development

By offering accurate personalized radiation dose prescriptions and monitoring systems may help to reduce the incidence and severity of radiation-induced pneumonitis and carditis in individual patients. To achieve this goal, it requires a highly integrated approach that combines diagnostic testing by means of imaging-based analysis of cellular signaling pathways, measurement of circulating or secreted biomarkers, and assessment of genomic and proteomic anomalies. Such an imaging agent should be designed to identify tumor location, severeness of cardiopulmonary injuries, radiotherapy dose, chemotherapy doses, and select patient for pathway-directed therapy. Integrated analysis of circulating phenotype pro-inflammatory cytokines, genotype SNV arrays, and cellular imaging could enhance the ability to predict which patients will develop RT-induced cardiopulmonary toxicity.

Traditional means of diagnosing pneumonitis include imaging (e.g., roentgenology or CT); blood tests; pulmonary function testing (with an oximeter or spirometer); bronchoscopy; or surgical lung biopsy (53). However, improvements in cancer diagnosis, prognostication, treatment planning, and treatment monitoring have been greatly enhanced by the development of radionuclide-labeled compounds specifically targeted to tumors. Positron emission tomography/computed tomography (PET/CT) or single photon emission computed tomography/computed tomography (SPECT/CT) are used to map the location and concentrations of these compounds (54–56). In current practice, PET and SPECT gamma cameras are fused with CT scanners to enhance the sensitivity of these imaging methods for quantifying radiolabeled compounds *in vivo* in real time. The addition of CT to PET and SPECT allows better delineation of tumor volumes *via* multiple slices (by CT) and serial imaging (by PET and SPECT); also, the anatomic and morphologic detail revealed by CT improves the ability to evaluate anatomic changes induced by the therapy. Although CT and MRI can provide considerable anatomic information about the location and extent of tumors, these imaging modalities cannot adequately distinguish invasive lesions from edema, radiation necrosis, shifts in tumor grading during treatment, or gliosis. Therefore, CT, MRI, and ultrasonography can be helpful in estimating prognosis but do not provide information on the functional status of the target, which limits their effectiveness for evaluating treatment outcomes. Moreover, the “gold standard” for determining treatment endpoints remains the molecular and histologic analysis of biopsy samples; supplementation of these findings with information on tumor metabolic activity by PET or SPECT can improve the localization and characterization of tumors. The use of image-guided treatment approaches in parallel with CT or MRI hybrid instrumentation would provide a more comprehensive picture of response to treatment in individual patients.

The radiopharmaceuticals used for PET/CT and SPECT/CT show high specific activities and have little to no detectable pharmacologic effects because they are created by nuclear transformation and use carrier-free forms of isotopes. Use of these radiotracers can provide important information on a variety of processes, including vascular angiogenesis (i.e. integrin, VEGF, EGFR) (57–60), hypoxia (61–63), apoptosis (64, 65), biomarkers (i.e. fibroblast activation protein, PSA) (66–69), and cellular signaling and transcriptional activity (70–72). Overall, PET/CT and SPECT/CT imaging agents thus enable the comprehensive characterization necessary for determining optimal therapeutic dosing, regulatory warning in drug-induced adverse event, differential diagnosis between inflammation/infection and recurrence, sensitivity vs resistance to treatments, the grade of tumors, and the ability to predict which patients will response to a given treatment.

#### 3.4.2 Imaging glycolysis pathways: Limitations of FDG-PET/CT

Functional cellular imaging in oncology has been focused on the identification of specific markers and the application of these markers to evaluate responses to RT or chemoradiation therapy. One such marker, the glucose analogue FDG, is used to assess uptake by glucose transporter and measure glucose metabolism. FDG is considered a “gold standard” radiopharmaceutical for PET and PET/CT. Visualization of FDG facilitates the detection of sites and extent of inflammation and infection as well as unsuspected distant metastases. FDG has also been used as an indirect measure of the glycolysis pathway (the conversion of glucose to lactate to produce adenosine triphosphate [ATP] for energy) and the hexokinase biosynthesis pathway (the conversion of glucose to intermediates that influence protein glycosylation), which both contribute to glucose metabolism and influence immune system function by prompting proliferation and activation of T cells. FDG uptake has been used to correlate recurrence and survival after treatment with high-dose proton therapy and chemotherapy in NSCLC (73). FDG has been used to manage symptoms of myocardial toxicity such as in distinguishing right from left ventricular dysfunction in myocarditis (74, 75).

^18^F-FDG-PET scanning can also aid in lung cancer staging, as it can visualize the extent of the primary tumor (76, 77) and the presence of mediastinal-node or systemic metastases. FDG-PET is also helpful for better defining radiation target volumes and for identifying patients with subclinical systemic disease who would be best served by palliative therapy. In locally advanced NSCLC, FDG PET/CT-based adaptive RT can be useful for quantifying reductions in target volume, which would allow the radiation dose to be escalated to the smaller target, and minimized to surrounding normal tissues, for the remainder of the treatment (78–81). However, FDG has some disadvantages. Although FDG PET/CT is sensitive for detecting inflammation, FDG is not disease-specific, thus, FDG produced false-positive in the differential diagnosis between inflammation/infection and tumor recurrence. Also, FDG tends to accumulate in the pericardium, pleura, salivary glands, eyelids, muscles, bone marrow, spleen, lymph nodes, joints, and major blood vessels (81, 82). With high background in these tissues, FDG produced false-negative results.

Optimal treatment planning also depends on the accurate staging and re-staging of intrathoracic (mediastinal) nodal status in NSCLC. For example, patients with NSCLC with no evidence of involvement of the ipsilateral mediastinal or subcarinal (N2) lymph nodes may be able to undergo surgical resection. Although the noninvasive nature of FDG-PET/CT is a major advantage for staging mediastinal disease, FDG is less optimal for detecting malignancy in normal‐sized lymph nodes and for ruling out malignancy in patients with coexisting inflammatory or infectious diseases (83–86). Although FDG-PET/CT can be used as a complement to examinations that show inconclusive results, its effectiveness and clinical benefit for patients with lymph node metastasis may be limited owing to its poor ability to distinguish inflammation from tumor recurrence. This feature may also be related to the subcellular distribution of FDG in the mitochondria and cytosol of cells (87, 88) and its inability to affect downstream transcriptional pathways. FDG complicates the distinction between lesions and background signal, which produces false-negative in myocardial imaging and false-positive in tumor staging and re-staging. These features, combined with an overall lack of specificity as a biomarker, impede the usefulness of FDG for identifying therapeutic response.

#### 3.4.3 Hexosamine biosynthetic pathways (HBP) systems imaging: Tracers beyond FDG

Glycolysis is upregulated in inflammation and cancer. Its metabolic pathways also contribute substrates for lipogenesis and protein glycosylation by producing acetyl-CoA, meaning that other tracers can complement glycolysis-based PET imaging. Examples of such tracers include radiolabeled acetate and choline, for imaging elevated glycolysis and lipogenesis; radiolabeled misonidazole, fluoroazomycin arabinoside, pimonidazole, and integrins can visualize tissue hypoxia and angiogenesis during periods of elevated glycolysis. However, these agents, like FDG, cannot distinguish inflammation/infection from tumor recurrence. Thus, the ability to image steps in the glycosylation and hexosamine biosynthetic pathways may be a more effective alternative for the diagnosis and assessment of DNA-damage response in lung tumors as well as RT-induced cardiopulmonary toxicities.

Two sets of glucose transporters are involved in this process, sodium-coupled glucose transporters and glucose transporter facilitators (88). Under normal conditions, 95% of the energy produced by cells occurs through glycolysis, which takes place in the cytosol; FDG, glucose, and glucosamine all rely on glucose transporter 1 (GLUT1) 1 and GLUT3 in glucose pathway-directed systems. Notably, cells subjected to stress (as occurs in cancer) require greater amounts of glucose to sustain energy production, which prompts a shift from glycolysis to glycosylation in HBP systems (89). In the HBP, glutamine:fructose-6-phosphate amidotransferase (GFAT) uses the amide group of glutamine to convert fructose 6-phosphate to glucosamine 6-phosphate; hence the formation of hexosamine products requires a supply of both glucose and glutamine (90, 91). However, a subsequent product of this pathway, N-acetylglucosamine enters the cells directly and is phosphorylated at positions 1 and 6 immediately, as opposed to free glucosamine or glutamine, which is followed by phosphorylation and then acetylation. Phosphorylated N-acetylglucosamine interacts with uridine diphosphate (UDP) to form UDP−N-acetylglucosamine, followed by the activation of NFAT (nuclear factor of activated T cells) and *O*-linked *N*-acetylglucosamine transferase (OGT). The dynamic glycosylation of serine or threonine residues on nuclear and cytosolic proteins by OGT is abundant in all multicellular eukaryotes. OGT participates in post-translational modification of a large number of nucleocytoplasmic proteins (92–95). OGT activity is exquisitely responsive to intracellular UDP−N-acetylglucosamine and UDP concentrations, which are in turn highly sensitive to glucose concentrations and other stimuli. This phenomenon is dynamic and often occurs in response to different stimuli (stress, heat) in its ability to form an UDP−N-acetylglucosamine-NfKb conjugate for internalization by the cell nucleus (92). In the cell nucleus, the ubiquitous transcription factor Sp1 is extensively modified by OGT. Sp1 becomes hyperglycosylated in response to hyperglycemia or elevated glucosamine (83). Moreover, O-linked N-acetylglucosamine glycosylation is also linked to DNA chromosomal X-linked intellectual disability (96).

Analysis of SNV arrays has helped to clarify the crosstalk among cellular pathways of genes involved in inflammatory processes. For example, OGT regulates IL-6/STAT3 signaling pathways and interacts with nuclear proteins (glycosylation), which is thought to contribute to numerous cellular processes including cell metabolism, proliferation, and inflammation in lung cancer (97, 98). Elevated OGT levels also correlate with increased pro-inflammatory IL-17A cytokine secretion by murine and human CD4^+^ T cells (99).

The metabolic activity of tumor cells, as qualitatively measured by FDG-PET, has been linked with increases in the amount of glucose membrane transporters and the activity of the principal enzymes controlling the glycolytic pathways. However, both glucosamine and glucose share the same pathway(s). Although glucose shares only 4% of the hexosamine biosynthetic pathway, glucosamine has been shown to share approximately 96% of both the glycolytic/citric acid cycle (i.e., tricarboxylic acid or Krebs cycle) and the hexosamine pathways. Because glucosamine is involved not only in the hexosamine pathway but also in DNA proliferative activity, it becomes an attractive imaging agent for differential diagnosis in cancer.

**Figure 4:**
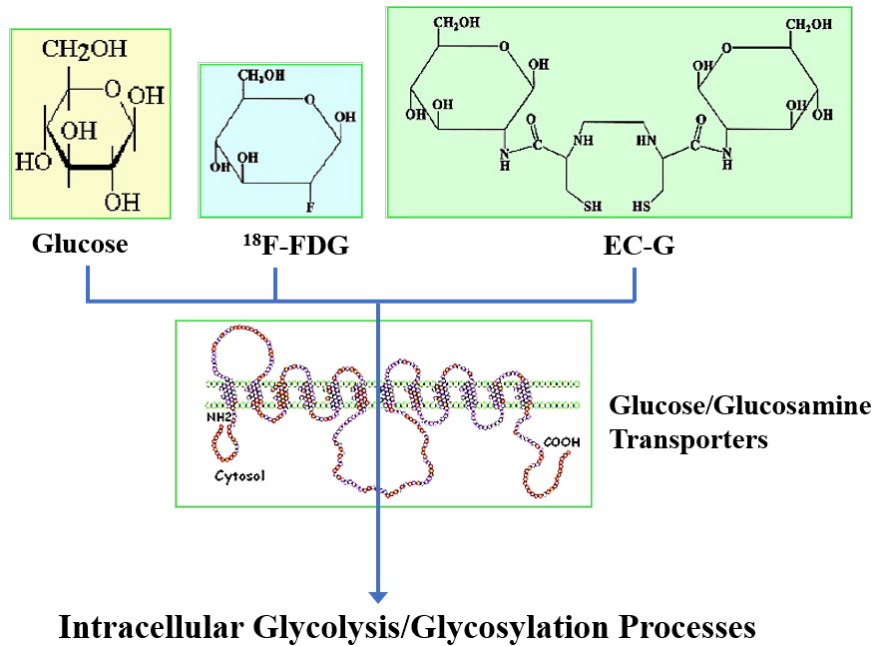
Structures of fluorodeoxyglucose (FDG), glucosamine and ethylenedicysteine-glucosamine (EC-G).

**Figure 5:**
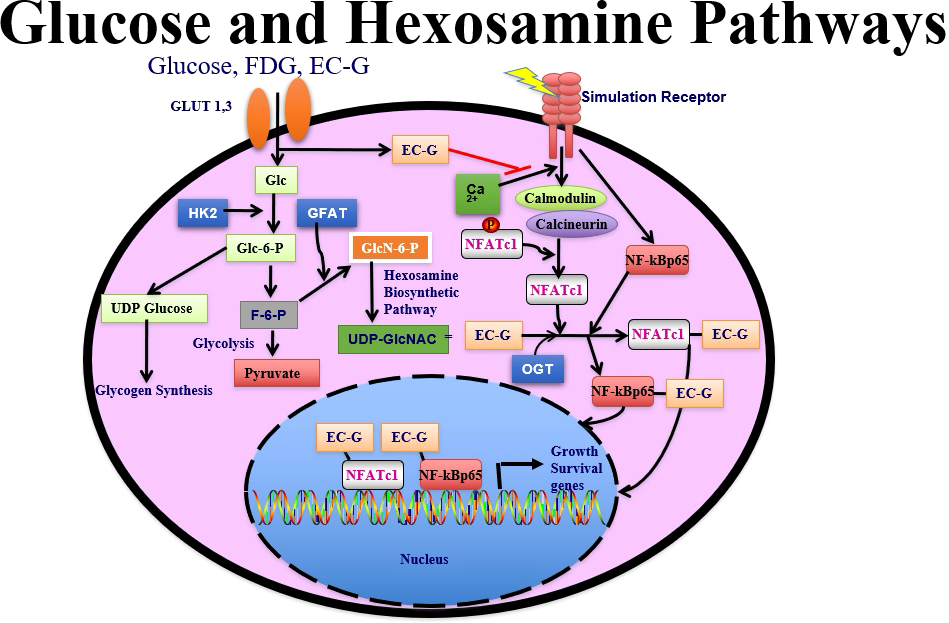
FDG is involved in glycolysis. EC-G has downstream to OGT glycosylation pathways. ^99m^Tc-EC-G measures cell cycle progression in cancer.

To assess N-acetylglucosamine pathways in HBP systems, several chelators have been conjugated to glucosamine to trace glucose and glucosamine transport systems by SPECT/CT. These chelator-glucosamine conjugates include ^99m^Tc-diethylenetriamine pentaacetate-glucosamine (DTPA-glucosamine) (100, 101), ^99m^Tc-ethylenedicysteine-glucosamine (EC-G) (102–105) and ^68^Ga-EC-G (106). These conjugates mimic the structure of N-acetylglucosamine. ^99m^Tc-DTPA-glucosamine had fast clearance in animal studies, its appearance in tumors was low (98). EC-G has two glucosamine moieties in the molecule (**Figure 4**). ^99m^Tc-EC-G was able to measure DNA proliferation in lung cancer cells and image tumors in animal models (105, 106). During inflammatory processes, expression of glucose, glutamine, and GFAT is high to produce higher levels of N-acetylglucosamine. Clinical studies revealed that ^99m^Tc-EC-G was safe and had favorable radiation dosimetry in NSCLC patients (104). ^99m^Tc-EC-G was able to distinguish tumor from inflammation due to its downstream post-transcriptional HBP with DNA proliferation (104–108) (**Figure 5**). ^99m^Tc-EC-G was not inferior to FDG for imaging in patients with NSCLC (102). The HBP is also activated under conditions of cellular oxidative stress such as myocardial ischemia. ^99m^Tc-EC-G has been shown to enter the pathway and provide a means to image a prior episode of myocardial ischemia in animal models (109). Consequently, ^99m^Tc-EC-G, a tumor-specific biomarker, may offer an alternative as a SPECT/CT agent for assessing dose-related RT-induced cardiopulmonary toxicities in NSCLC.

## 4 Future perspectives

The benefits of high-dose RT for early-stage NSCLC have been well established, but, as discussed above, come at the potential risk of radiation-induced toxicity, chiefly pneumonitis and carditis. Radiation-induced cardiopulmonary toxicities were dose-limiting factors. One way of minimizing the risk of these potentially deadly side effects is the use of traditional (anatomic) imaging modalities to delineate the target volume (i.e., the gross tumor volume versus clinic tumor volume), and develop treatment plans based on those images to ensure that radiation to the target is maximized and that to the surrounding normal tissues is minimized. Use of serial imaging before each radiation fraction (as opposed to obtaining images only once, before treatment is begun) can help to address DNA-damage changes in tumor size and location as the treatment progresses, to continue to meet target and tissue dose limits. The addition of cellular imaging, for example by PET/CT or SPECT/CT, can help to refine target delineation by including information on cellular function status. Current knowledge of imaging pathways for glycosylation by ^99m^Tc-EC-G is beyond glucose glycolysis by FDG as ^99m^Tc-EC-G applies to the detection of tumor DNA proliferation, myocardial ischemia and minimization of RT-induced pneumonitis and myocardial injuries.

In addition to functional imaging, analysis of variants in the human genome can provide a reference for comparison with sequencing data. SNVs are the simplest form of DNA variation among individuals. SNVs can influence not only the amino acid sequences in the resultant proteins but also genetic stability, non-coding genes, the microenvironment, and the formation of intracellular fused proteins. Identification of new SNVs and the resultant allele(s) associated with known polymorphisms by direct DNA sequencing may reveal clues as to how an individual’s genetic makeup can predispose them to develop pneumonitis or carditis. As one example, functional SNVs in genes coding for inflammatory cytokines can influence the functions of those cytokines, which in turn can influence the host reaction to malignancy, infection, and inflammation. Immune cytokines such as interleukin-6 (IL-6) and interleukin (IL-17) also participate in T-cell–mediated inflammatory processes. Using an integrated approach in which traditional imaging and dose-volume analyses are combined with analysis of immune cytokine patterns, SNV sequences, and shifts in HBP systems imaging will help to customize adaptive dose escalation to residual active tumor regions and, ultimately, to reduce the incidence of RT-induced pneumonitis and carditis.

## 5 Conclusions

Radiotherapy for locally advanced NSCLC confers a risk of pneumonitis and myocardial injury, which directly affect treatment outcomes, including quality of life and overall survival. Proton therapy can minimize RT-induced pneumonitis and myocardial injury, which can confer benefit in response and prolong survival in comparison with conventional X-ray radiotherapy. Use of radiolabeled ligands, radiolabeled antibodies, and signal transduction agents in imaging has opened a new era for evaluating pathway-directed processes. The application of hybrid imaging instrument (PET and SPECT hybrid with CT and MRI) can improve the differential diagnosis by enhancing specificity, correcting for attenuation, and [sharpening] localization. The focus of cellular imaging agent has shifted to predicting therapeutic response, differential diagnosis, and monitoring tumor response. Both FDG and ^99m^Tc-EC-G are suitable for initial diagnosis (baseline) prior to RT-induced cardiopulmonary toxicities. The areas of tumor and predisposed tissue injuries in non-small cell lung cancer (NSCLC) could be quantified by the uptake of radiopharmaceuticals. Owing to the lack of specificity by FDG, ^99m^Tc-EC-G may be an alternative radiopharmaceutical for repeated imaging of tumor DNA-damage response *via* HBP at post-treatment. The dose-volume histogram metrics measured by ^99m^Tc-EC-G with the correlative analyses of IL-6, IL-17, OGT and SNVs of HBP have potential to overcome heterogeneity of the tumor, and tumor location as well as derive an accurate radiation dose limits to reduce the risk of RT-induced cardiopulmonary toxicities. The integrated assessments of cytokine phenotype patterns, genotype SNVs and imaging findings will help to personalize radiation therapy in terms of radiation dose, reduce adverse events by detecting time to cardiopulmonary toxicities in response to radiation, and optimize treatment outcomes.

## Author contributions

ZL designed the content of manuscript. YM-S and DY searched articles relating to the subject and wrote the manuscript under ZL and TI’s supervision. ZL and TI helped in final editing. All authors contributed to the article and approved the submitted version.

## Glossary

**Table:**

AS-PCR	allele-specific polymerase chain reaction
CPK	creatine phosphokinase
CT	computed tomography
2D	2-dimensional
DTPA	diethylenetriamine pentaacetate
ECHO	echocardiogram
ECM	extracellular matrix
EGFR	epidermal growth factor receptor
FDG	^18^F-fluorodeoxyglucose
FGF	fibroblast growth factor
GFAT	glutamine:fructose-6-phosphate amidotransferase
GLUT	glucose transporter
HBP	hexosamine biosynthetic pathways
HIF-1	Hypoxia-inducible factor-1
ICAM	intercellular adhesion molecule
IGF	insulin-like growth factor
IL	interleukin
IMRT	intensity-modulated radiation therapy
IFN	interferons
JAK-STAT	Janus kinases-signal transducer and activator of transcription proteins
KGF	Keratinocyte Growth Factor
LVSF	left ventricular shortening fraction
mPCR-RETINA	multiplex PCR-based real-time invader assay
mTOR	mechanistic target of rapamycin
NADPH	Nicotinamide adenine dinucleotide phosphate
NFAT	nuclear factor of activated T cells
NF-κB	nuclear factor kappa-light-chain-enhancer of activated B cells
NGS	next generation sequence
NSCLC	non-small cell lung cancer
OGT	*O*-linked *N*-acetylglucosamine transferase
PDGF	platelet-derived growth factor
PET	positron emission tomography
RFLP	restriction fragment length polymorphism
RNS	reactive nitrogen species
ROS	reactive oxygen species
RT	radiation therapy
SABR	stereotactic ablative body radiation
SBRT	stereotactic body radiation therapy
SCLC	small cell lung cancer
SNP	single nucleotide polymorphism
SNV	single nucleotide variants
Sp-1	transcription factor specificity protein 1
SPECT	single photon emission computed tomography
SRS	stereotactic radiosurgery
^99m^Tc-EC-G	^99m^Tc-ethylenedicysteine-glucosamine
TGF	transforming growth factor
TNF-α	tumor necrosis factor-alpha
UDP-GlcNAc	uridine diphosphate-N-acetyl glucosamine
VCAM	vascular cell adhesion molecule
VEGF	vascular endothelial growth factor
VMAT	volumetric modulated arc therapy
